# Active lifestyles related to excellent self-rated health and quality of life: cross sectional findings from 194,545 participants in The 45 and Up Study

**DOI:** 10.1186/1471-2458-13-1071

**Published:** 2013-11-13

**Authors:** Richard R Rosenkranz, Mitch J Duncan, Sara K Rosenkranz, Gregory S Kolt

**Affiliations:** 1 School of Science and Health, University of Western Sydney, Sydney, Australia; 2 Department of Human Nutrition, Kansas State University, Manhattan, KS, USA; 3 Institute for Health and Social Science Research, Centre for Physical Activity Studies, Central Queensland University, Rockhampton, Australia

**Keywords:** Physical activity, Sedentary behavior, Sitting time, Sedentary lifestyle, Salutogenic, Health promotion, Adult, Older adult, Successful aging, Quality of life

## Abstract

**Background:**

Physical activity and sitting time independently contribute to chronic disease risk, though little work has focused on aspirational health outcomes. The purpose of this study was to examine associations between physical activity, sitting time, and excellent overall health (ExH) and quality of life (ExQoL) in Australian adults.

**Methods:**

The 45 and Up Study is a large Australian prospective cohort study (n = 267,153). Present analyses are from 194,545 participants (48% male; mean age = 61.6 ± 10.7 yrs) with complete baseline questionnaire data on exposures, outcomes, and potential confounders (age, income, education, smoking, marital status, weight status, sex, residential remoteness and economic advantage, functional limitation and chronic disease). The Active Australia survey was used to assess walking, moderate, and vigorous physical activity. Sitting time was determined by asking participants to indicate number of hours per day usually spent sitting. Participants reported overall health and quality of life, using a five-point scale (excellent—poor). Binary logistic regression models were used to analyze associations, controlling for potential confounders.

**Results:**

Approximately 16.5% of participants reported ExH, and 25.7% reported ExQoL. In fully adjusted models, physical activity was positively associated with ExH (AOR = adjusted odds ratio for most versus least active = 2.22, 95% CI = 2.20, 2.47; *P*_trend_ < 0.001) and ExQoL (AOR for most versus least active = 2.30, 95% CI = 2.12, 2.49; *P*_trend_ < 0.001). In fully adjusted models, sitting time was inversely associated with ExH (AOR for least versus most sitting group = 1.13, 95% CI = 1.09, 1.18; *P*_trend_ < 0.001) and ExQoL (AOR for least versus most sitting group = 1.13, 95% CI = 1.10, 1.17; *P*_trend_ < 0.001). In fully adjusted models, interactions between physical activity and sitting time were not significant for ExH (*P* = 0.118) or ExQoL (*P* = 0.296).

**Conclusions:**

Physical activity and sitting time are independently associated with excellent health and quality of life in this large diverse sample of Australian middle-aged and older adults. These findings bolster evidence informing health promotion efforts to increase PA and decrease sitting time toward the achievement of better population health and the pursuit of successful aging.

## Background

Worldwide, nations are preparing for the demands of an aging population, and this entails dealing with challenges of maintaining health, functional capacity, and wellbeing [1]. Focusing on relevant lifestyle behaviors is an important consideration for preventing or delaying chronic disease and improving health [1-3]. An emerging body of literature indicates that the lifestyle behaviors of physical activity and time spent sitting independently contribute to health outcomes such as chronic disease morbidity and mortality risk [4].

Regularly engaging in moderate-to-vigorous physical activity has been shown to reduce the risk of all-cause mortality, cardiovascular mortality, cancer mortality, stroke, heart disease, breast cancer, colon cancer, and other undesirable health outcomes [5]. Over the past decade, however, research on the health impacts of sedentary behavior (time spent at low levels of energy expenditure while in a sitting posture) has expanded rapidly [4]. High volumes of time spent sitting are associated with an increased risk of all-cause mortality [6-10], cardiovascular disease mortality [8], type 2 diabetes mellitus [11-15], and other diseases or conditions [15-17] when adjusting for participation in moderate-to-vigorous intensity physical activity. Therefore, insufficient moderate-to-vigorous physical activity and sitting time may be distinct influences on poor health.

Compared to abundant literature on risk factors for disease and poor health, research focusing on the influence of physical activity and sitting time on more aspirational health-related outcomes is much less common [5,18-20]. Successful aging has been described as a multidimensional intersection, where not only the avoidance of disease and disability are found, but also where high cognitive and physical function and engagement with life conjoin [21]. The focus on such aspirational outcomes represents a “salutogenic” approach to health promotion [22], rather than the traditional disease prevention approach. This salutogenic orientation is instructive for determining influences on aspirational levels of health and well-being. Aspirational positively framed messages may be more effective for motivating healthful behavior in some segments of the population, compared to focusing on the avoidance of chronic disease, which is often an abstract possibility many years away [23].

Despite the aging population and widespread problem of physical inactivity, there has been limited use of successful aging or salutogenic approaches to frame positive health messages toward motivating active lifestyles. This study examines both self-reported health and quality of life status as they are useful health outcomes and are predictive of more objective health indicators [24]. To investigate whether higher levels of physical activity and lower levels of sitting time were positively associated with excellent health and quality of life, we utilized self-reported data from a large sample of middle-aged and older Australian men and women, and we statistically adjusted for a range of associated covariates and potential confounding variables in the analyses.

## Methods

### The 45 and Up Study

The 45 and Up Study is a large ongoing Australian prospective cohort study that began with a baseline sample of 267,153 men and women from New South Wales, the most populous state in Australia. A detailed description of The 45 and Up Study has been published previously [25]. The 45 and Up Study baseline [26] data provide information on a wide range of health-related variables. Participants were randomly sampled from the Medicare Australia (national health insurance) database between February 2006 and December 2008. All adults who were aged 45 years and over and who were currently residing in NSW at the time of recruitment were eligible for inclusion in the Study. Participants who completed a mailed baseline questionnaire and provided their signed consent for participation in the baseline questionnaire and long-term follow-up were included in the Study [25]. The University of NSW Human Research Ethics Committee provided approval for The 45 and Up Study and analysis of the baseline questionnaire data (approval number 05035). The University of Western Sydney Human Research Ethics Committee granted reciprocal institutional ethics approval for use of the baseline questionnaire data in the current study (UWS Protocol number H8793).

### Participants

Participants were a subgroup (n = 194,545) of the total baseline sample of 267,153 men and women enrolled in The 45 and Up Study as of December 2009 (18% response rate). The 45 and Up Study sample was intended to be a large heterogeneous sample of Australian adults, though not necessarily a true representation of the Australian adult population. The present study’s sample included all participants aged 45–106 years with non-missing data on self-rated overall health, quality of life, physical activity, sitting time, and covariates and potential confounding variables (age, household income, educational qualification, smoking status, marital status, weight status, sex, residential remoteness and economic advantage, functional limitation, and number of chronic diseases). Thus, the final sample included 194,545 residents (48% male) of New South Wales, aged 45–106 years (mean ± SD = 61.6 ± 10.7 yrs), from The 45 and Up Study baseline dataset. All participant data, except region of residence (Medicare records), originated from responses to a self-administered paper questionnaire that was completed and returned by postal mail.

### Physical activity and sitting time

The Active Australia Survey (AAS) [27], was used to measure physical activity in The 45 and Up Study baseline questionnaire. This instrument has previously demonstrated acceptable test-retest reliability [28] and validity [29]. On the questionnaire, participants were asked to indicate their participation in three types of physical activity over the previous week– “walking continuously, for at least 10 minutes (for recreation or exercise or to get to or from places)”; “vigorous physical activity (that made you breathe harder or puff and pant, like jogging, cycling, aerobics, competitive tennis, but not household chores or gardening)”; and “moderate physical activity (like gentle swimming, social tennis, vigorous gardening or work around the house)” – by recording the total duration and the total number of times they participated in each [27]. For this study, total minutes spent in the queried physical activities was used to determine physical activity levels, with vigorous physical activity time multiplied by two, for double weighting [27]. In accordance with previous research using this dataset [15] physical activity time was divided into five categories of total minutes per week, as follows: zero mins; low active (1–149 mins); sufficiently active (150–299 mins); highly active (300–539 mins); and very highly active (540+ mins).

Total sitting time was determined by asking participants to report total hours per day usually spent sitting. In accordance with previous research arising from the 45 and Up baseline dataset [15], sitting time was divided into four categories of 0 to <4 hours; 4 to <6 hours; 6 to <8 hours; and 8 hours or more of sitting time per day for analysis. Although reliability and validity of this sitting time questionnaire item has not been formally assessed, the item is analogous to the sitting time item used in the International Physical Activity Questionnaire (IPAQ), shown to have acceptable reliability and validity [30]. Atkin et al. [31] support the use of single-item questionnaires in epidemiological research, when the primary requirements of such research consist of the ability to rank levels of health-related variables within the sample.

### Self-rated health and quality of life

Self-rated overall health was assessed with the following question, “In general, how would you rate your overall health?” Five response options included: excellent; very good; good; fair; or poor. For the current study, self-rated health was dichotomized as excellent or not excellent (including very good; good; fair; and poor). Self-rated quality of life was assessed with the following question, “In general, how would you rate your quality of life?” Five response options included: excellent, very good, good, fair, or poor. Self-rated quality of life was dichotomized as excellent or not excellent.

### Covariates and potential confounding variables

To control potential confounding in analyses, covariates included age group, household income, educational qualification, residential remoteness, residential economic advantage, marital status, smoking status, weight status, number of chronic diseases, and level of functional limitation. Participants indicated their age in years, categorized into five age groups: 45 to 54; 55 to 64; 65 to 74; 75 to 84; 85 and up. Highest educational qualification was categorically self-reported, including: no school certificate; school certificate; high school certificate; trade or apprenticeship; certificate or diploma; or university degree. Participants indicated whether they had ever been a regular smoker; smoking status was dichotomously categorized as “ever” or “never.”

Marital status was self-reported according to six categories, reduced to a dichotomous variable for analysis as married (including married; de facto/living with a partner) or not married (single; widowed; divorced; separated). Residential remoteness and residential economic advantage were determined based on the mean Accessibility Remoteness of Australia Plus score for participant home address postcode. Five residential remoteness categories included: major city, inner regional, outer regional, remote, or very remote area. Four residential economic advantage categories included: least, mid to low, mid to high, and most economic advantage. Annual household income was categorized for analysis as less than $10,000, $10,000-$29,999, $30,000-$49,999, $50,000-$69,999, or $70,000 or more.

Weight status was determined from self-reported height and weight to calculate body mass index (km/m^2^), using WHO classifications [32] to determine underweight (<18.50 km/m^2^), normal weight (18.50–24.99 km/m^2^), overweight (25.00–29.99 km/m^2^) and obese (≥30.00 km/m^2^) categories. Functional limitation status was determined using the Medical Outcomes Study Physical Functioning (MOS-PF) scale, which assesses the extent to which an individuals’ health limits their ability to perform daily functional activities [33]. The MOS-PF has demonstrated good test-retest reliability and content validity as a measure of physical functioning [34]. Based on a 100-point scale, functional limitation scores were categorized as: no functional limitation (100), minor limitation (95–99), moderate limitation (85–94), or severe limitation (0–84).

Participants reported whether they had ever been told by a doctor that they have skin cancer, melanoma, breast cancer, other cancer, heart disease, prostate cancer, enlarged prostate, high blood pressure, stroke, diabetes, blood clot, asthma, hay fever, depression, anxiety, or Parkinson’s disease. Chronic diseases were categorized for analysis as: none, one, or two or more chronic diseases.

### Statistical methods

Data from The 45 and Up Study baseline dataset were analyzed using SPSS 19.0 software (SPSS Inc. Chicago, IL USA) for both descriptive and inferential statistics. Crude odds ratios (OR) and adjusted odds ratios (AOR) with 95% confidence intervals (CI) were calculated to assess the association between exposures and the outcome variables of excellent health and excellent quality of life using separate binary logistic regression models. Potential confounders were added to the model in groups of demographic and physical health variables, and interaction between physical activity and sitting time was examined via an interaction term. Logistic regression models were mutually adjusted for categories of physical activity or sitting time (model 1); followed by additional adjustment for categories of age, household income, educational qualification, smoking status, marital status, weight status, sex, and remoteness and economic advantage of residential area (model 2); and lastly, a fully adjusted model included additional adjustment for categories of physical limitation and chronic diseases (model 3). To examine the consistency of relationships between active lifestyle variables and health-related outcomes, follow-up logistic regression analyses were used, stratified by age group, sex, household income, weight status, and self-reported ancestry (Australian or not). A final fully adjusted binary logistic regression (model 3) was used to examine excellent self-rated health and quality of life by 20 combination categories of five physical activity and four sitting levels. A significance level of alpha = 0.05 was used for all analyses.

## Results

Approximately 16.5% of the sample reported excellent overall health and 25.7% reported excellent quality of life (Table 1). The unadjusted associations between socio-demographic and lifestyle factors are provided in Table 2. For both excellent quality of life and excellent health, significant bivariate relationships were found for sitting time (inverse), physical activity, sex, marital status, age (inverse), income, education, residential economic advantage, residential remoteness (inverse), smoking (inverse), chronic disease (inverse), functional limitation, (inverse) and weight status (inverse from normal weight).

**Table 1:** Self-rated health and quality of life status prevalence and active lifestyles in the 45 and Up Study baseline sample (N =194,545)

		**Total sample**		**Very highly active**^**#**^		**Sitting**^**‡**^	
				**≥540mins/week**		**0 to <4 hrs/day**	
		**N**	**%**	**N**	**%**	**N**	**%**
Self-rated health							
	Excellent	31,738	16.5	17,763	56.0	9,214	29.0
	Very Good	73,852	38.4	35,158	47.6	19,494	26.4
	Good	62,417	32.5	24,127	38.7	15,373	24.6
	Fair	20,629	10.7	6,201	30.1	4,452	21.6
	Poor	3,672	1.9	723	19.7	698	19.0
Self-rated quality of life							
	Excellent	48,787	25.7	25,679	52.6	13,531	27.7
	Very Good	73,141	38.5	32,775	44.8	18,597	25.4
	Good	50,328	26.5	19,260	38.3	12,498	24.8
	Fair	14,929	7.9	4,573	30.6	3,358	22.5
	Poor	2,792	1.5	677	24.2	563	20.2

^#^Refers to those within each level of health and quality of life who were very highly active;

^‡^Refers to those within each level of health and quality of life who sat 0 to <4 hrs/day.

**Table 2:** Bivariate associations between participant characteristics and self-rated health and quality of life status in the 45 and Up Study baseline sample (N =194,545)

				**Excellent health**	**Excellent quality of life**
		**N**	**%**	OR* (95% CI)	OR* (95% CI)
Sex					
	Male†	95,242	48.4	1.00	1.00
	Female	100,293	51.6	1.38 (1.34—1.41)	1.31 (1.28—1.33)
Sitting time per day (hours)					
	≥8†	50,402	25.9	1.00	1.00
	6 to <8	38,806	19.9	0.99 (0.95—1.02)	1.03 (0.99—1.06)
	4 to <6	55,435	28.5	1.01 (0.97—1.04)	1.05 (1.02—1.08)
	0 to <4	49,902	25.7	1.23 (1.19—1.27)	1.18 (1.15—1.22)
Physical activity per week (minutes)					
	0†	7,624	3.9	1.00	1.00
	1 to 149	30,086	15.5	1.60 (1.44—1.77)	1.75 (1.61—1.90)
	150 to 299	30,524	15.7	2.52 (2.27—2.79)	2.65 (2.44—2.87)
	300 to 539	41,413	21.3	3.45 (3.12—3.82)	3.34 (3.09—3.61)
	≥540	84,898	43.6	4.48 (4.06—4.95)	4.05 (3.75—4.38)
Age group (years)					
	45 to 54†	62,038	31.933.8	1.00	1.00
	55 to 64	65,796	33.8	0.84 (0.82—0.86)	0.94 (0.92—0.97)
	65 to 74	40,444	20.8	0.58 (0.56—0.60)	0.69 (0.67—0.71)
	75 to 84	22,037	11.3	0.32 (0.30—0.33)	0.37 (0.35—0.39)
	85 and older	4,230	2.2	0.20 (0.17—0.23)	0.21 (0.19—0.23)
Household income					
	less than $10 K†	9,699	5.0	1.00	1.00
	$10 to <30 K	45,958	23.6	1.18 (1.09—1.28)	1.33 (1.25—1.43)
	$30 to <50 K^#^	62,408	32.1	2.09 (1.94—2.26)	2.38 (2.23—2.53)
	$50 to <70 K	22,037	11.7	2.59 (2.38—2.80)	2.93 (2.74—3.14)
	$70 K or more	53,803	27.7	3.83 (3.55—4.14)	4.37 (4.09—4.66)
Residential remoteness					
	Major city†	88,446	45.5	1.00	1.00
	Inner regional area	68,785	35.4	0.92 (0.89—0.94)	1.02 (1.00—1.04)
	Outer regional area	33,743	17.3	0.86 (0.83—0.89)	0.98 (0.95—1.01)
	Remote area	3,259	1.7	0.72 (0.65—0.80)	0.88 (0.81—0.96)
	Very remote area	312	0.2	0.67 (0.48—0.94)	0.96 (0.74—1.25)
Residential economic advantage					
	Least advantaged†	48,713	25.0	1.00	1.00
	Mid—low advantage	45,756	23.5	1.11 (1.07—1.15)	1.07 (1.04—1.10)
	Mid—high advantage	48,894	25.1	1.25 (1.20—1.29)	1.15 (1.12—1.18)
	Most advantaged	51,182	26.3	1.68 (1.62—1.74)	1.47 (1.43—1.51)
Educational qualification					
	No school certificate†	18,646	9.6	1.00	1.00
	School certificate	40,685	20.9	1.59 (1.49—1.68)	1.57 (1.50—1.65)
	High school certificate	19,588	10.1	2.01 (1.88—2.14)	1.92 (1.82—2.03)
	Trade/apprenticeship	21,426	11.0	1.44 (1.34—1.53)	1.50 (1.42—1.58)
	Certificate/diploma	43,251	22.2	2.20 (2.08—2.33)	2.22 (2.12—2.33)
	University degree	50,949	26.2	3.34 (3.16—3.53)	3.29 (3.14—3.44)
Smoking status					
	Never regular smoker†	110,119	56.6	1.00	1.00
	Ever regular smoker	84,426	43.4	0.67 (0.65—0.68)	0.72 (0.71—0.74)
Weight status					
	Underweight	2,404	1.2	0.65 (0.58—0.72)	0.59 (0.53—0.65)
	Normal weight†	71,499	36.8	1.00	1.00
	Overweight	77,684	39.9	0.59 (0.57—0.60)	0.76 (0.74—0.78)
	Obese	42,958	22.1	0.24 (0.23—0.25)	0.46 (0.45—0.48)
Chronic diseases					
	None†	71,221	36.6	1.00	1.00
	One	67,886	34.9	0.59 (0.57—0.60)	0.75 (0.73—0.76)
	Two or more	55,438	28.5	0.23 (0.22—0.24)	0.43 (0.42—0.44)
Functional limitation					
	No limitation†	66,747	34.3	1.00	1.00
	Minor limitation	34,012	17.5	0.40 (0.38—0.41)	0.64 (0.62—0.65)
	Moderate limitation	36,819	18.9	0.20 (0.19—0.20)	0.39 (0.38—0.40)
	Severe limitation	56,967	29.3	0.05 (0.05—0.06)	0.12 (0.11—0.12)
Marital status					
	Not married, divorced, separated †	45,492	23.4	1.00	1.00
	Married, de facto	149,053	76.6	1.33 (1.29—1.37)	1.82 (1.77—1.86)

†Reference category; *Unadjusted odds ratio; #includes those selecting “prefer not to answer”.

### Sitting time

Table 3 presents the odds of excellent overall self-rated health and excellent quality of life by categories of sitting time. In model 1, the lowest sitting time category showed a significant positive association in log-odds of excellent health and quality of life, relative to the highest sitting time category of ≥8 hours per day (AOR for lowest versus highest category = 1.09; 95% CI = 1.05, 1.13; *P*_trend_ < 0.001) and excellent quality of life (AOR for lowest versus highest category = 1.07; 95% CI = 1.04, 1.10; *P*_trend_ < 0.001).

**Table 3:** Odds of excellent overall health and quality of life by sitting time and physical activity (N =194,545)

	**Excellent health**		
	**Adjusted OR**^**a **^**(95% CI)**	**Adjusted OR**^**b **^**(95% CI)**	**Adjusted OR**^**c **^**(95% CI)**
Sitting time (hrs/day)			
≥8†	1.00	1.00	1.00
6 to <8	0.90 (0.87—0.94)	1.05 (1.01—1.09)	1.04 (0.99—1.08)
4 to <6	0.90 (0.87—0.93)	1.10 (1.06—1.14)	1.05 (1.01—1.09)
0 to <4	1.09 (1.05—1.13)	1.26 (1.21—1.30)	1.13 (1.09—1.18)
Physical activity (mins/week)			
0†	1.00	1.00	1.00
1 to 149	1.61 (1.44—1.78)	1.28 (1.15—1.42)	1.10 (0.98—1.23)
150 to 299	2.53 (2.28—2.81)	1.73 (1.56—1.92)	1.37 (1.23—1.53)
300 to 539	3.47 (3.14—3.84)	2.28 (2.06—2.53)	1.69 (1.51—1.88)
≥540	4.51 (4.08—4.98)	3.20 (2.89—3.54)	2.22 (2.00—2.47)
**Excellent quality of life**			
	**Adjusted OR**^**a**^** (95% CI)**	**Adjusted OR**^**b**^** (95% CI)**	**Adjusted OR**^**c**^** (95% CI)**
Sitting time (hrs/day)			
≥8†	1.00	1.00	1.00
6 to <8	0.95 (0.92—0.98)	1.09 (1.05—1.12)	1.08 (1.04—1.11)
4 to <6	0.95 (0.93—0.98)	1.14 (1.11—1.18)	1.10 (1.07—1.14)
0 to <4	1.07 (1.04—1.10)	1.23 (1.19—1.27)	1.13 (1.10—1.17)
Physical activity (mins/week)			
0†	1.00	1.00	1.00
1 to 149	1.75 (1.61—1.90)	1.44 (1.33—1.57)	1.29 (1.19—1.41)
150 to 299	2.65 (2.45—2.87)	1.92 (1.77—2.09)	1.62 (1.49—1.76)
300 to 539	3.35 (3.09—3.62)	2.35 (2.17—2.55)	1.86 (1.71—2.02)
≥540	4.05 (3.75—4.38)	3.06 (2.82—3.31)	2.30 (2.12—2.49)

†Reference category.

^a^Model 1. Odds for sitting time and moderate to vigorous physical activity, mutually adjusted for each other.

^b^Model 2. Same adjustment from model 1, additional adjustment for categories of age, household income, educational qualification, smoking status, marital status, weight status, sex, and remoteness and economic advantage of residential area.

^c^Model 3. Same adjustment from model 3, additional adjustment for categories of functional limitation and number of chronic diseases.

In the fully adjusted model 3, all categories of sitting time displayed significantly higher log-odds of excellent self-rated health, relative to the category sitting ≥8 hours per day. The category reporting the lowest amount of sitting was 13% more likely to report excellent health (AOR for lowest versus highest category = 1.13; 95% CI = 1.09, 1.18; *P*_trend_ < 0.001) compared to those sitting ≥8 hours per day. All categories of sitting time showed higher log-odds of excellent quality of life, compared with the highest sitting category. The category reporting least sitting time was 13% more likely to report excellent quality of life (AOR for lowest versus highest category = 1.13; 95% CI = 1.10, 1.17; *P*_trend_ < 0.001) compared to those sitting ≥8 hours per day.

### Physical activity

Table 3 presents the odds of excellent overall health and excellent quality of life by five categories of physical activity. In model 1, all categories of physical activity above zero minutes showed significantly higher log-odds of excellent health and quality of life, relative to those reporting zero minutes. The most physically active group was more than four times as likely to report excellent health (AOR for highest versus lowest category = 4.51; 95% CI =4.08, 4.98; *P*_trend_ < 0.001) and excellent quality of life (AOR for highest versus lowest category = 4.05; 95% CI = 3.75, 4.38; *P*_trend_ < 0.001), compared to the least physically active.

In the fully adjusted model 3, all categories of physical activity above zero minutes displayed significantly higher log-odds of excellent health and quality of life, relative to the lowest physical activity category. The most physically active category was twice as likely to report excellent health (AOR for highest versus lowest category = 2.22; 95% CI = 2.00, 2.47; *P*_trend_ < 0.001) and twice as likely to report excellent quality of life (AOR for highest versus lowest category = 2.30; 95% CI = 2.12, 2.47; *P*_trend_ < 0.001), compared to the least physically active.

### Stratified analyses

A series of fully adjusted binary logistic regressions (model 3), used to examine variations across demographic variables, are presented in Additional file 1: Tables S1-S4. In age-stratified analyses, the relationship between physical activity and excellent health was strongest for the oldest age group (AOR for highest versus lowest category = 4.54; 95% CI = 1.78, 11.56). For the weight status-stratified analyses, the relationship between physical activity and excellent health was strongest for the underweight group (AOR for highest versus lowest category = 6.60; 95% CI = 1.56, 28.01). For both health and quality of life, across all other strata of age, sex, household income, ancestry, and weight status, adjusted odds ratios for most physically active versus least active category centered just over 2.0, ranging from 1.58—2.80. For sitting time, adjusted odds ratios for lowest sitting time versus highest centered just over 1.0, ranging from 0.88—1.31 for both health and quality of life outcomes, across all other strata.

### Interaction of physical activity and sitting time

In logistic regression model 1, examining relationships between physical activity, sitting time, and excellent health and quality of life, the physical activity and sitting time interaction terms were significant (*P* = 0.001 for health; *P* = 0.003 for quality of life). These interactions were not significant, however, in the fully adjusted models (*P* = 0.118 for health; *P* = 0.296 for quality of life).

**Figure 1:**
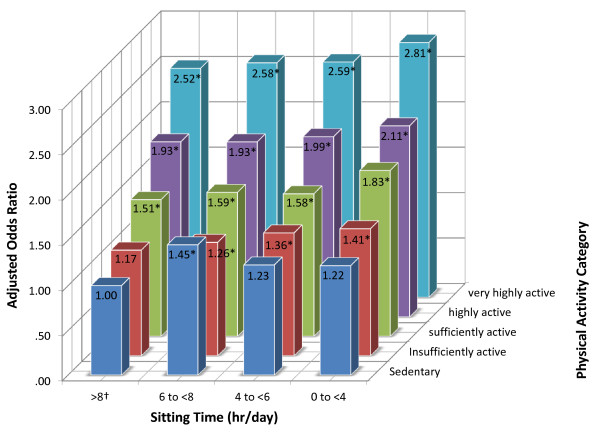
**Odds of excellent self-rated health by category of sitting time and physical activity level.** N = 194,545. †Reference category is those with lowest physical activity level who sit 8 or more hours per day. ^#^Model adjusted for categories of age, household income, educational qualification, smoking status, marital status, weight status, sex, and remoteness and economic advantage of residential area, functional limitation, and number of chronic diseases. *p < 0.05; All AOR > 1.25 Significantly different from 1.00.

Figure 1 graphically displays the fully adjusted (model 3) log-odds of excellent health by 20 combinations of physical activity and sitting time. In this figure, the reference category is the most inactive group: those reporting zero minutes of physical activity and eight or more hours per day of sitting. The most physically active group was nearly three times as likely to report excellent health compared to the least active group (AOR for very highly active and sitting 0 to <4 hours per day versus zero minutes and sitting ≥8 hours per day = 2.81; 95% CI = 2.33, 3.38, *P*_trend_ < 0.001).

**Figure 2:**
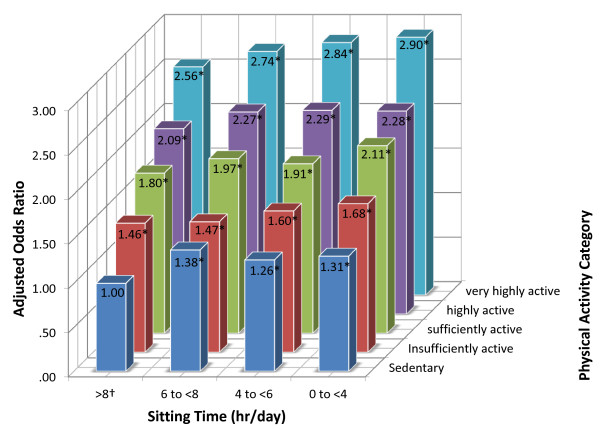
**Odds of excellent quality of life by category of sitting time and physical activity level.** N = 194,545. †Reference category is those with lowest physical activity level who sit 8 or more hours per day. ^#^Model adjusted for categories of age, household income, educational qualification, smoking status, marital status, weight status, sex, and remoteness and economic advantage of residential area, functional limitation, and number of chronic diseases. *p < 0.05; All AOR > 1.25 Significantly different from 1.00.

Figure 2 depicts the fully adjusted odds (model 3) of excellent quality of life by combinations of physical activity and sitting time. The most physically active group was nearly three times as likely to report excellent quality of life compared to the least active group (AOR for very highly active and sitting 0 to <4 hours per day, versus zero minutes of physical activity and sitting ≥8 hours per day = 2.90; 95% CI = 2.52, 3.34, *P*_trend_ < 0.001).

## Discussion

Stemming from a salutogenic approach and positive health message framework, we sought to investigate whether higher levels of physical activity and lower levels of sitting time were positively associated with excellent health and quality of life. This study’s main finding was that both physical activity and sitting time were independently associated with excellent health and excellent quality of life, showing physical activity was the stronger influence of the two. These associations were attenuated when controlling for key demographic influences of age, household income, education, and weight status, but remained statistically significant and likely of public health significance. The associations were further attenuated when controlling for key health-related influences of chronic disease and physical limitation, but remained significant and likely meaningful with regard to the health of this population. Although there was some indication of interaction between physical activity and sitting time, the interactions were not statistically significant in fully adjusted models. Therefore, the final models for both excellent health and quality of life appear to reflect two independent main effects of physical activity and sitting time.

The likelihood of reporting excellent health and quality of life was substantially higher for individuals with an active lifestyle, as compared to their less active counterparts. Physical activity and sitting time both showed dose–response patterns of influence on the odds of excellent health and quality of life, when controlling for other key behavioral and environmental influences. In our stratified analyses, the robustness of relationships between the health-related outcomes and physical activity and sitting time was remarkable. Across most demographic strata, lower levels of sitting time generally showed small positive relationships with the aspirational outcomes of excellent health and quality of life. Physical activity too, was mostly consistent across strata, manifesting a strong salutogenic factor in this study. These patterns did not hold, however, for adults aged 85 and older (n = 4,230), or for underweight adults (n = 2,404) who constituted a small minority of this sample, and who have different profiles of health, quality of life, and risk of chronic disease than the majority of the population.

Conceptually, self-rated health reflects a global assessment related to functioning and presence or absence of diseases or symptoms, while health-related quality of life reflects the discrepancy between actual and desired functional status, and the overall impact of health on well-being [24]. Although the proportion of our sample reporting excellent quality of life was about 9% higher than the proportion reporting excellent health, the associations between lifestyle behaviors and these two health-related outcomes were strikingly similar. For most analyses, participants who were highly active or very highly active (300 minutes or more per week) were about twice as likely to report excellent health and excellent quality of life, as compared to their least active counterparts. Within the highly active or very highly active categories, the likelihood of excellent health or quality of life was higher for those reporting less sitting time per day, on the order of about a 20-30% difference between those sitting most and those sitting least each day at this level of physical activity. The influence of sitting, however, was clearly less of an influence than physical activity for likelihood of excellent health and quality of life.

### Integration with previous studies

The present study’s results are similar to those of Davies et al. [18], who examined associations between physical activity and screen time on health-related quality of life. Their study found that the combination of no physical activity and high screen time (e.g., watching television—typically sedentary behavior) was related to poorer quality of life in their sample of Australian adults. Our study differed from theirs, however, by focusing on excellent health and quality of life in a larger sample of middle-aged and older adults, and examining sitting time rather than screen time. We also were able to statistically adjust for additional influences such as functional limitation, residential economic advantage and remoteness.

Kerr et al. [35] found that higher physical activity levels were significantly related to greater self-rated health in a sample of American adults, aged 66 years and up, but these researchers did not include sitting time in their analyses. Vallance et al. [19], however, recently showed a relationship between sitting and health-related quality of life in men aged 55 years and up, when adjusting for physical activity. In their study, those who reported sitting the least time had better physical, mental, and global health, compared to those who sat the most, but these relationships only held for weekend sitting time.

The present study aligns with previous studies showing that sitting time and physical activity are independently related to health-related outcomes when both lifestyle components are studied concurrently [8-10,15]. Similar to previous studies, our results showed some indication that the influence of physical activity and sitting time may interact [7,10,18], but the interaction was not robust, and was attenuated and insignificant in the fully adjusted model.

The present findings suggest that physical activity and sitting time may each present a potential avenue to increase the likelihood of excellent health and quality of life, but that the combination of more physical activity and less sitting time may offer the greatest potential for attaining excellent health and quality of life. If such findings are borne out by additional research studies, positive health messages could be used to motivate populations to adopt more active lifestyles.

Although our findings align with much of the previous literature showing better health associated with more physical activity and less sitting time, not all studies have supported such relationships. In particular, Herber-Gast et al. [36] recently reported no association between sitting time and incidence of cardiovascular disease when controlling for physical activity and other relevant demographic and lifestyle factors in a longitudinal analysis of middle-aged Australian women. These authors found no interaction between physical activity and sitting time related to cardiovascular disease. The divergence from our findings may be due to differences in focusing on disease more than health, focusing solely on women, or possibly in differing measures of physical activity and sitting time.

In work more relevant to the salutogenic model, Vallance et al. [19] similarly did not show an association between sitting time and physical health, mental health, or global health when examining weekday sitting separately from weekend sitting. Trinh and colleagues [37] also reported few significant associations between sitting time and quality of life in cancer survivors. Södergren and colleagues [3] reported that although leisure time physical activity was associated with good health in adults aged 55–65 years, sitting time was not significantly related to measures of good health. The lack of association in these previous studies may represent unknown moderation by demographic variables, or may stem from residual confounding by physical activity level, age, work type and status, or other relevant influences on sitting time and health. Particularly relevant to our study, the impact of physical activity was stronger than that of sitting time, and controlling for this key lifestyle variable along with other important potential confounding variables in smaller datasets with more limited power or greater variability may partially explain null findings in previous studies [3]. Our observed fully adjusted odds ratios for sitting time were small in magnitude for the lowest versus highest sitting categories, and this magnitude of association is unlikely to be statistically significant in much smaller datasets.

### Areas for further study

Despite the wealth of research on physical activity and health-related outcomes, the investigation of sitting time has only recently proliferated, and most of this work has focused on associations with chronic disease and mortality. Both physical activity and sitting time are linked with a wide range of health-related outcomes [5,38]. Both of these active lifestyle components can change energy expenditure, which is related to reduced risk of morbidity and mortality in much of the literature [5,38]. Yet, like the wide variety of biological and psychological mechanisms thought to play a role for physical activity, there is likely more to sitting’s influence on health and quality of life than energy expenditure alone.

Spending long periods in occupational sitting is associated with overall fatigue, musculoskeletal pain, and poor health in data from interviews with office workers [39]. In the ergonomics literature, sitting is linked to one of the most prevalent chronic conditions, low back pain [40], frequently associated with disability [41]. Thus, prolonged bouts of sitting daily may potentially feature prominently in a downward spiral of decreased mobility, physical function, physical fitness, engagement with life, physical activity, and eventually greater risk of chronic disease [42], but much more work is required to examine these possibilities and their temporal sequence.

Beyond the focus on chronic diseases [15], indices of cardiometabolic health [12], and mortality [9], research on active lifestyles stemming from being physically active and limiting prolonged sitting has been moving into studies of mental wellbeing [43-45], cognitive function [38] and health-related quality of life [3,19]. More work is required in this area to determine associations with mood, energy level, sexual and neurological functioning, sleep, and activities of daily living, among many important health-related outcomes.

Further prospective studies should investigate active lifestyles and successful aging from a salutogenic approach [22], with a deliberate focus on the components of physical and cognitive functioning and engagement with life, plus avoidance of disease [21]. More evidence from longitudinal prospective research is needed, including the continued use of The 45 and Up Study data as further time points become available. Armed with epidemiological evidence, further work in etiology and lifestyle determinants of successful aging can include experimental studies, and public health intervention work can examine positive message framing to promote excellent health and quality of life through more active lifestyles. Public health efforts should also address a need for guidelines or recommendations for limiting prolonged sitting or reductions in this sedentary behavior, to accompany physical activity guidelines.

### Strengths and limitations

A major strength of the current study is the use of a very large heterogeneous sample of Australian adults, with a diversity of age, socioeconomic status, residential characteristics, and other demographic and lifestyle influences on health and quality of life. An additional strength of this study was our ability to stratify analyses and statistically control for potential confounding variables that may limit or distort findings from such observational studies. Lastly, our study was novel in the use of a salutogenic approach to frame positive health messages toward motivating healthful active lifestyle behaviors and successful aging.

Opposite these strengths stand the major limitation of cross-sectional analysis of these baseline data from the ongoing longitudinal 45 and Up Study. Cross-sectional analysis precludes any causal implications of the observed relationships. Further studies with this 45 and Up Study and others will be better able to show temporality of association, particularly as follow-up data become available in the near future. Self-report measures of sitting time and physical activity may be susceptible to various types of bias, but our measures of physical activity and sitting time were sufficient for ranking individuals within an epidemiological dataset for analysis [31]. Also, our measures of overall health and quality of life were self-reported, and therefore inherently subjective. Despite this, patient-reported health status has been shown to be an independent predictor of subsequent mortality, cardiovascular events, hospitalization, and costs of care [24]. Therefore, such measures are highly relevant in public health research.

## Conclusions

Physical activity and sitting time are independently associated with excellent health and quality of life in this large diverse sample of Australian middle-aged and older adults. The present study’s findings bolster evidence to inform efforts to increase moderate-to-vigorous physical activity and decrease time spent sitting toward the achievement of better population health and pursuit of successful aging. Public health efforts can use the accumulating body of evidence on active lifestyles to develop guidelines or recommendations for sitting, in addition to those of physical activity. Public health efforts could consider framing positive health messages and health promotion interventions aimed at achievement of higher levels of health and quality of life through moving more and sitting less to motivate middle-aged and older adults to improve their lifestyles.

## Abbreviations

OR: Odds ratio; AOR: Adjusted odds ratio; CI: Confidence interval; ExH: Excellent self-rated health; ExQoL: Excellent self-rated quality of life; BMI: Body mass index; NSW: New South Wales; UWS: University of Western Sydney; AAS: Active Australia Survey; WHO: World Health Organization; MOS-PF: Medical outcomes study physical functioning scale; IPAQ: International physical activity questionnaire; SD: Standard deviation.

## Competing interests

The authors declare that they have no competing interests.

## Authors’ contributions

RRR conceived of the study, participated in the design and coordination of the study, performed the statistical analysis and drafted the manuscript. SKR participated in the design and coordination of the study, assisted with the statistical analysis and contributed to the preparation of the manuscript. MJD participated in the design and coordination of the study, assisted with statistical analysis and contributed to the preparation of the manuscript. GSK participated in the design and coordination of the study and contributed to the preparation of the manuscript. All authors read and approved the final manuscript.

## Pre-publication history

The pre-publication history for this paper can be accessed here:

http://www.biomedcentral.com/1471-2458/13/1071/prepub

## Supplementary Material

Additional file 1: Table S1 Age-stratified odds of excellent overall health and quality of life by sitting time and physical activity (N =194,545) **Table S2.** Sex-stratified and ancestry-stratified odds of excellent overall health and quality of life by sitting time and physical activity (N =194,545). **Table S3.** Household-income-stratified odds of excellent overall health and quality of life by sitting time and physical activity (N =194,545). **Table S4.** Body-mass-index-stratified odds of excellent overall health and quality of life by sitting time and physical activity (N =194,545). Click here for file
