# Supplementary material for: Active lifestyles related to excellent self-rated health and quality of life: cross sectional findings from 194,545 participants in The 45 and Up Study
Source: BMC Public Health. 2013 Nov 13;13:1071. doi: 10.1186/1471-2458-13-1071 (PMC3831595; doi:10.1186/1471-2458-13-1071)
Supplement: Additional file 1: Table S1 — Age-stratified odds of excellent overall health and quality of life by sitting time and physical activity (N =194,545) Table S2. Sex-stratified and ancestry-stratified odds of excellent overall health and quality of life by sitting time and physical activity (N =194,545). Table S3. Household-income-stratified odds of excellent overall health and quality of life by sitting time and physical activity (N =194,545). Table S4. Body-mass-index-stratified odds of excellent overall health and quality of life by sitting time and physical activity (N =194,545). [file 1471-2458-13-1071-S1.doc]

Supplementary Table 1. Age-stratified odds of excellent overall health and quality of life by sitting time and physical activity (N =194,545)

|  | Excellent health adjusted* OR (95%CI) | | | |  |
| --- | --- | --- | --- | --- | --- |
|  | 45—54 years | 55—64 years | 65—74 years | 75—84 years | 85+ years |
| Sitting time (hrs/day) |  |  |  |  |  |
| >8† | 1.00 | 1.00 | 1.00 | 1.00 | 1.00 |
| 6 to <8 | 1.05 (0.98—1.11) | 1.01 (0.95—1.08) | 1.03 (0.92—1.14) | 1.00 (0.84—1.19) | 0.89 (0.57—1.39) |
| 4 to <6 | 1.04 (0.98—1.11) | 1.03 (0.97—1.10) | 1.00 (0.90—1.10) | 1.15 (0.98—1.35) | 1.09 (0.73—1.62) |
| 0 to <4 | 1.16 (1.10—1.23) | 1.12 (1.05—1.19) | 1.07 (0.97—1.19) | 1.09 (0.92—1.29) | 0.95 (0.61—1.47) |
|  |  |  |  |  |  |
| Physical activity (mins/week) |  |  |  |  |  |
| 0† | 1.00 | 1.00 | 1.00 | 1.00 | 1.00 |
| 1 to 149 | 1.06 (0.89—1.26) | 1.09 (0.89—1.33) | 0.95 (0.72—1.25) | 1.11 (0.77—1.61) | 3.86 (1.50—9.92) |
| 150 to 299 | 1.34 (1.13—1.60) | 1.38 (1.14—1.68) | 1.21 (0.92—1.59) | 1.26 (0.87—1.83) | 3.23 (1.19—8.74) |
| 300 to 539 | 1.81 (1.53—2.15) | 1.60 (1.32—1.93) | 1.19 (0.91—1.56) | 1.68 (1.17—2.41) | 7.09 (2.74—18.32) |
| >540 | 2.49 (2.11—2.94) | 2.11 (1.75—2.54) | 1.58 (1.22—2.05) | 1.77 (1.25—2.51) | 4.54 (1.78—11.56) |
|  |  | | | |  |
|  | Excellent quality of life adjusted* OR (95%CI) | | | |  |
|  | 45—54 years | 55—64 years | 65—74 years | 75—84 years | 85+ years |
| Sitting time (hrs/day) |  |  |  |  |  |
| >8† | 1.00 | 1.00 | 1.00 | 1.00 | 1.00 |
| 6 to <8 | 1.06 (1.00—1.12) | 1.07 (1.01—1.13) | 1.08 (0.99—1.17) | 1.06 (0.93—1.20) | 0.84 (0.59—1.19) |
| 4 to <6 | 1.08 (1.03—1.14) | 1.11 (1.06—1.17) | 1.04 (0.97—1.12) | 1.08 (0.96—1.22) | 1.17 (0.86—1.59) |
| 0 to <4 | 1.10 (1.04—1.15) | 1.13 (1.08—1.20) | 1.12 (1.03—1.21) | 1.13 (1.00—1.29) | 0.88 (0.62—1.25) |
|  |  |  |  |  |  |
| Physical activity (mins/week) |  |  |  |  |  |
| 0† | 1.00 | 1.00 | 1.00 | 1.00 | 1.00 |
| 1 to 149 | 1.21 (1.05—1.39) | 1.21 (1.04—1.40) | 1.32 (1.06—1.64) | 1.48 (1.12—1.95) | 2.01 (1.18—3.44) |
| 150 to 299 | 1.43 (1.25—1.64) | 1.56 (1.34—1.81) | 1.70 (1.37—2.10) | 1.81 (1.37—2.39) | 2.31 (1.31—4.09) |
| 300 to 539 | 1.72 (1.51—1.97) | 1.78 (1.54—2.06) | 1.71 (1.39—2.11) | 2.11 (1.61—2.77) | 2.45 (1.39—4.31) |
| >540 | 2.18 (1.91—2.48) | 2.14 (1.85—2.47) | 2.18 (1.78—2.68) | 2.35 (1.81—3.07) | 2.94 (1.74—4.96) |
|  |  |  |  |  |  |

†Reference category

*Odds for sitting time and moderate to vigorous physical activity, mutually adjusted for each other, additional adjustment for categories of household income, educational qualification, smoking status, marital status, weight status, sex, and remoteness and economic advantage of residential area, categories of functional limitation and number of chronic diseases

Supplementary Table 2. Sex-stratified and ancestry-stratified odds of excellent overall health and quality of life by sitting time and physical activity (N =194,545)

|  | Excellent health adjusted* OR (95%CI) | | | |  |  |
| --- | --- | --- | --- | --- | --- | --- |
|  | Males | Females | Australian Ancestry | Not Australian Ancestry |  |  |
| Sitting time (hrs/day) |  |  |  |  |  |  |
| >8† | 1.00 | 1.00 | 1.00 | 1.00 |  |  |
| 6 to <8 | 1.01 (0.95—1.07) | 1.06 (1.00—1.12) | 1.03 (0.98—1.09) | 1.04 (0.98—1.10) |  |  |
| 4 to <6 | 1.03 (0.98—1.09) | 1.07 (1.02—1.12) | 1.04 (0.99—1.10) | 1.05 (0.99—1.11) |  |  |
| 0 to <4 | 1.13 (1.07—1.20) | 1.15 (1.09—1.20) | 1.12 (1.06—1.18) | 1.14 (1.09—1.21) |  |  |
|  |  |  |  |  |  |  |
| Physical activity (mins/week) |  |  |  |  |  |  |
| 0† | 1.00 | 1.00 | 1.00 | 1.00 |  |  |
| 1 to 149 | 1.03 (0.87—1.21) | 1.15 (0.99—1.34) | 1.05 (0.89—1.23) | 1.14 (0.97—1.34) |  |  |
| 150 to 299 | 1.28 (1.09—1.51) | 1.44 (1.24—1.67) | 1.24 (1.06—1.44) | 1.51 (1.29—1.76) |  |  |
| 300 to 539 | 1.63 (1.39—1.92) | 1.72 (1.48—1.99) | 1.56 (1.34—1.81) | 1.80 (1.55—2.10) |  |  |
| >540 | 2.21 (1.89—2.59) | 2.21 (1.92—2.55) | 2.01 (1.73—2.34) | 2.42 (2.08—2.81) |  |  |
|  |  | | | |  |  |
|  |  | | | | | |
|  | Excellent quality of life adjusted* OR (95%CI) | | | | | |
|  | Males | Females | Australian Ancestry | Not Australian Ancestry |  |  |
| Sitting time (hrs/day) |  |  |  |  |  |  |
| >8† | 1.00 | 1.00 | 1.00 | 1.00 |  |  |
| 6 to <8 | 1.07 (1.02—1.12) | 1.08 (1.03—1.13) | 1.09 (1.04—1.14) | 1.06 (1.01—1.11) |  |  |
| 4 to <6 | 1.11 (1.06—1.16) | 1.10 (1.05—1.15) | 1.10 (1.05—1.15) | 1.10 (1.05—1.15) |  |  |
| 0 to <4 | 1.14 (1.09—1.20) | 1.13 (1.08—1.18) | 1.14 (1.09—1.19) | 1.13 (1.08—1.18) |  |  |
|  |  |  |  |  |  |  |
| Physical activity (mins/week) |  |  |  |  |  |  |
| 0† | 1.00 | 1.00 | 1.00 | 1.00 |  |  |
| 1 to 149 | 1.22 (1.07—1.38) | 1.36 (1.21—1.53) | 1.22 (1.08—1.37) | 1.37 (1.21—1.56) |  |  |
| 150 to 299 | 1.52 (1.34—1.72) | 1.70 (1.51—1.91) | 1.52 (1.35—1.71) | 1.71 (1.51—1.94) |  |  |
| 300 to 539 | 1.79 (1.59—2.02) | 1.91 (1.70—2.14) | 1.70 (1.52—1.90) | 2.02 (1.79—2.28) |  |  |
| >540 | 2.29 (2.03—2.57) | 2.30 (2.06—2.58) | 2.07 (1.85—2.31) | 2.54 (2.25—2.86) |  |  |
|  |  |  |  |  |  |  |

†Reference category

*Odds for sitting time and moderate to vigorous physical activity, mutually adjusted for each other, additional adjustment for categories of age, household income, educational qualification, smoking status, marital status, weight status, sex, and remoteness and economic advantage of residential area, categories of functional limitation and number of chronic diseases

Supplementary Table 3. Household-income-stratified odds of excellent overall health and quality of life by sitting time and physical activity (N =194,545)

|  | Excellent health adjusted* OR (95%CI) | | | | |
| --- | --- | --- | --- | --- | --- |
|  | Less than $10K  n | $10K to <30K | $30K to <50K# | $50K to <$70K | $70K or more |
| Sitting time (hrs/day) |  |  |  |  |  |
| >8† | 1.00 | 1.00 | 1.00 | 1.00 | 1.00 |
| 6 to <8 | 0.91 (0.67—1.22) | 1.07 (0.95—1.20) | 1.05 (0.97—1.13) | 1.04 (0.94—1.17) | 1.03 (0.97—1.09) |
| 4 to <6 | 1.02 (0.78—1.32) | 1.14 (1.03—1.27) | 1.06 (0.99—1.14) | 1.04 (0.94—1.15) | 1.02 (0.96—1.08) |
| 0 to <4 | 1.15 (0.89—1.49) | 1.20 (1.07—1.33) | 1.16 (1.08—1.24) | 1.09 (0.98—1.21) | 1.12 (1.05—1.19) |
|  |  |  |  |  |  |
| Physical activity (mins/week) |  |  |  |  |  |
| 0† | 1.00 | 1.00 | 1.00 | 1.00 | 1.00 |
| 1 to 149 | 1.07 (0.63—1.82) | 0.98 (0.75—1.27) | 1.25 (1.02—1.53) | 1.07 (0.78—1.47) | 1.05 (0.86—1.28) |
| 150 to 299 | 1.37 (0.80—2.33) | 1.21 (0.94—1.57) | 1.66 (1.37—2.02) | 1.30 (0.95—1.78) | 1.29 (1.06—1.56) |
| 300 to 539 | 1.69 (1.02—2.83) | 1.40 (1.09—1.79) | 1.84 (1.52—2.23) | 1.73 (1.27—2.35) | 1.68 (1.39—2.03) |
| >540 | 2.21 (1.35—3.60) | 1.76 (1.38—2.24) | 2.37 (1.96—2.85) | 2.17 (1.61—2.94) | 2.34 (1.94—2.82) |
|  |  | | | |  |
|  |  | | | |  |
|  | Excellent quality of life adjusted* OR (95%CI) | | | | |
|  | Less than $10K  n | $10K to <30K | $30K to <50K# | $50K to <$70K | $70K or more |
| Sitting time (hrs/day) |  |  |  |  |  |
| >8† | 1.00 | 1.00 | 1.00 | 1.00 | 1.00 |
| 6 to <8 | 1.04 (0.81—1.33) | 1.02 (0.93—1.12) | 1.10 (1.03—1.17) | 1.02 (0.93—1.11) | 1.08 (1.03—1.14) |
| 4 to <6 | 1.33 (1.07—1.65) | 1.06 (0.97—1.15) | 1.09 (1.03—1.16) | 1.08 (0.99—1.18) | 1.10 (1.04—1.15) |
| 0 to <4 | 1.31 (1.05—1.63) | 1.09 (1.01—1.19) | 1.13 (1.07—1.20) | 1.07 (0.98—1.16) | 1.13 (1.07—1.19) |
|  |  |  |  |  |  |
| Physical activity (mins/week) |  |  |  |  |  |
| 0† | 1.00 | 1.00 | 1.00 | 1.00 | 1.00 |
| 1 to 149 | 0.98 (0.65­—1.48) | 1.36 (1.11—1.67) | 1.40 (1.20—1.63) | 1.23 (0.97—1.57) | 1.19 (1.03—1.39) |
| 150 to 299 | 1.46 (0.97—2.20) | 1.82 (1.48—2.23) | 1.77 (1.52—2.05) | 1.57 (1.24—2.00) | 1.40 (1.21—1.63) |
| 300 to 539 | 1.87 (1.26—2.78) | 1.98 (1.62—2.41) | 2.00 (1.76—2.31) | 1.89 (1.50—2.40) | 1.62 (1.40—1.88) |
| >540 | 2.09 (1.43—3.06) | 2.40 (1.98—2.91) | 2.40 (2.08—2.77) | 2.15 (1.71—2.71) | 2.13 (1.85—2.46) |
|  |  |  |  |  |  |

†Reference category

*Odds for sitting time and moderate to vigorous physical activity, mutually adjusted for each other, additional adjustment for categories of age, educational qualification, smoking status, marital status, weight status, sex, and remoteness and economic advantage of residential area, categories of functional limitation and number of chronic diseases; ^#^includes those selecting “prefer not to answer”

Supplementary Table 4. Body-mass-index-stratified odds of excellent overall health and quality of life by sitting time and physical activity (N =194,545)

|  | Excellent health adjusted* OR (95%CI) | | | | |
| --- | --- | --- | --- | --- | --- |
|  | Normal Weight | Underweight | Overweight | Obese | |
| Sitting time (hrs/day) |  |  |  |  | |
| >8† | 1.00 | 1.00 | 1.00 | 1.00 | |
| 6 to <8 | 0.99 (0.94—1.05) | 1.13 (0.76—1.68) | 1.08 (1.01—1.15) | 1.03 (0.91—1.16) | |
| 4 to <6 | 1.00 (0.95—1.06) | 0.94 (0.65—1.36) | 1.08 (1.02—1.15) | 1.11 (0.99—1.23) | |
| 0 to <4 | 1.08 (1.02—1.14) | 1.03 (0.72—1.47) | 1.18 (1.11—1.25) | 1.20 (1.07—1.34) | |
|  |  |  |  |  | |
| Physical activity (mins/week) |  |  |  |  | |
| 0† | 1.00 | 1.00 | 1.00 | 1.00 | |
| 1 to 149 | 1.18 (0.99—1.42) | 3.48 (0.79—15.27) | 1.00 (0.84—1.20) | 1.10 (0.87—1.41) | |
| 150 to 299 | 1.47 (1.23—1.76) | 4.51 (1.03—19.75) | 1.30 (1.09—1.54) | 1.25 (1.00—1.60) | |
| 300 to 539 | 1.76 (1.48—2.10) | 4.19 (0.97—18.10) | 1.64 (1.38—1.95) | 1.52 (1.21—1.93) | |
| >540 | 2.40 (2.02—2.85) | 6.60 (1.56—28.01) | 2.09 (1.76—2.47) | 1.92 (1.53—2.41) | |
|  |  | | | | |
|  |  | | | | |
|  | Excellent quality of life adjusted* OR (95%CI) | | | | |
|  | Normal Weight | Underweight | Overweight | | Obese |
| Sitting time (hrs/day) |  |  |  | |  |
| >8† | 1.00 | 1.00 | 1.00 | | 1.00 |
| 6 to <8 | 1.05 (1.00—1.11) | 1.16 (0.82—1.63) | 1.10 (1.04—1.15) | | 1.07 (0.99—1.16) |
| 4 to <6 | 1.08 (1.02—1.13) | 0.96 (0.70—1.32) | 1.13 (1.08—1.19) | | 1.10 (1.02—1.18) |
| 0 to <4 | 1.10 (1.05—1.16) | 0.96 (0.70—1.32) | 1.15 (1.10—1.21) | | 1.17 (1.08—1.26) |
|  |  |  |  | |  |
| Physical activity (mins/week) |  |  |  | |  |
| 0† | 1.00 | 1.00 | 1.00 | | 1.00 |
| 1 to 149 | 1.40 (1.20—1.64) | 1.48 (0.63—3.45) | 1.21 (1.06—1.40) | | 1.30 (1.11—1.52) |
| 150 to 299 | 1.67 (1.44—1.95) | 2.26 (0.97—5.27) | 1.59 (1.39—1.83) | | 1.58 (1.35—1.86) |
| 300 to 539 | 1.94 (1.67—2.25) | 2.40 (1.04—5.50) | 1.83 (1.60—2.09) | | 1.79 (1.53—2.09) |
| >540 | 2.49 (2.15—2.88) | 2.80 (1.25—6.30) | 2.20 (1.93—2.50) | | 2.15 (1.88—2.49) |
|  |  |  |  | |  |

†Reference category

*Odds for sitting time and moderate to vigorous physical activity, mutually adjusted for each other, additional adjustment for categories of age, household income, educational qualification, smoking status, marital status, sex, and remoteness and economic advantage of residential area, categories of functional limitation and number of chronic diseases.
